# Ultrasound-Guided Upper Trunk Perineural Catheter for Shoulder Surgery: A Description of Catheter Technique

**DOI:** 10.7759/cureus.11095

**Published:** 2020-10-22

**Authors:** Richa Wardhan, Sindhuja R Nimma

**Affiliations:** 1 Anesthesiology, University of Florida, Gainesville, USA; 2 Anesthesiology, Mayo Clinic, Jacksonville, USA

**Keywords:** interscalene brachial plexus block, ultrasound guided blocks and vascular access, upper trunk block, reverse shoulder arthroplasty

## Abstract

Interscalene brachial plexus block is frequently utilized for anesthesia and analgesia of complex and painful shoulder surgeries. But unintentional phrenic nerve blockade is a bane to the existence of this technique. Single-injection upper trunk blockade has emerged as a promising approach that appears to preserve phrenic nerve function better than the interscalene approach. The purpose of this case series is to describe the sonoanatomy, technique, and utility of a continuous upper trunk block, not previously described in the literature.

## Introduction

The single-injection technique for blockade of the upper trunk of the brachial plexus was first brought to light by Burkett-St Laurent et al. in 2014 [1] followed by Aguirre et al. in 2016 [2]. The upper trunk block has been successfully demonstrated in cadavers, and two randomized control trials have further established the advantage of blocking the upper trunk over the conventional interscalene block in preserving the phrenic nerve function [3-5]. However, the continuous perineural catheter technique has not yet been promulgated in the literature. We present the case series of three patients who received upper trunk perineural catheters and describe our technique and experience in detail. All patients provided written HIPAA (Health Insurance Portability and Accountability Act of 1996) authorization to share their medical information and/or pictures to aid in preparation of this article.

## Case presentation

Case 1

A 77-year-old man was admitted for evaluation and treatment of complete tear of his infraspinatus and posterior labrum along with arthritis of the right shoulder. He underwent right reverse total shoulder arthroplasty, right biceps tenodesis, and right subscapularis tenotomy. The patient’s history was significant for chronic shoulder pain, lymphoma, and hypertension. He was given a physical status of American Society of Anesthesiologists (ASA) 3 and had a BMI of 27 kg/m^2^. He reported a pre-operative visual analog scale (VAS) pain score of 3-4 at rest. An upper trunk perineural catheter was placed preoperatively under sedation. A 10-mL bolus of 0.2% ropivacaine was administered, and a perineural pump (Curlin 6000™ infusion pump, Moog Medical Devices Group, Elma, NY, USA) was started at a rate of 5 mL/hour. Surgery was performed under general anesthesia, which was uneventful. The patient reported a VAS pain score of 0-2 immediately post-operatively. The morning of POD (post-operative day) 1, the patient reported a VAS score of 0/10 and requested removal of the nerve block catheter and was subsequently discharged home.

Case 2

A 72-year-old female was scheduled for a left rotator cuff repair ASA 3 and a BMI of 18 kg/m^2^. The patient reported a VAS pain score of 1 at rest. An upper trunk nerve catheter was placed and initiated with 10 mL of 0.2% ropivacaine with an infusion running at 5 mL/hour. In the recovery unit, the patient reported a VAS pain score of 0. The patient was discharged home on the same day with an ambulatory pain pump (ambIT® PIB-PCA, Summit Medical Products Inc., Alpharetta, GA, USA). She reported home VAS pain scores of 1-2 on PODs 1 and 2, and the catheter was removed by the patient on POD 3.

Case 3

A 44-year-old female presented with a proximal humerus fracture. She had a BMI of 23 kg/m^2^ and a past medical history of diabetes mellitus, gastroesophageal reflux disease, asthma, and rheumatoid arthritis. She underwent an open reduction and internal fixation of the right proximal humerus fracture. The pre-operative VAS score was 8-9 at rest.

The upper trunk catheter was placed preoperatively under sedation, and a bolus of 10 mL of 0.2% ropivacaine was administered. The pump was started at 5 mL/hour. The patient then underwent general anesthesia with no surgical complications. Immediately, in the recovery room, the patient reported a VAS score of 0. On POD 1, the patient had an allergic reaction to cefepime, resulting in a rash and shortness of breath. She was transferred to the ICU on 2L nasal cannula, and the nerve block was paused for 8 hours with no improvement in respiratory symptoms. There was no evidence of elevated hemidiaphragm on the ipsilateral side and/or pneumothorax. The perineural pump was restarted. On POD 2, the patient was discharged with a portable perineural pump, which was subsequently removed on POD 4.

## Discussion

Sonoanatomy of the upper trunk of brachial plexus

The C (cervical) 5 and C6 nerve roots lie close to their respective transverse processes as they enter the interscalene groove sandwiched between the anterior scalene and middle scalene muscles. The C6 nerve root then characteristically splits into two hypoechoic branches. The roots become increasingly superficial within the interscalene groove and eventually unite to form the upper trunk situated superior to the subclavian artery. At this stage, the hypoechoic nerve roots form a brighter more hyperechoic upper trunk, lying just below the prevertebral (deep cervical) fascia [1].

Description of technique

We used a high- or intermediate-frequency 6- to 15-MHz linear array transducer (X-Porte, Sonosite, Bothell, WA, USA) to provide adequate imaging. For best visualization and ergonomics, we recommend that the patient be placed in lateral position with the procedure side nondependent. Full aseptic precautions are maintained during block placement and catheter insertion. We prefer to use stimulating perineural catheters (Arrow Ultracath™ continuous nerve block kit, Telefex Incorporated, Wayne, PA, USA). The sheathed linear ultrasound probe is used to first identify the C5 and C6 nerve roots. This can be approached in two ways:

(1) Commencing the ultrasound scan in the supraclavicular fossa at the location of the supraclavicular brachial plexus, sliding the probe cephalad to identify the superior trunk, which is then seen arising from C5 and C6 nerve roots in the interscalene groove.

(2) Initiating the ultrasound scan at the level of the cricoid cartilage and identifying the C5 and C6 nerve roots, which is then seen sonoanatomically, coalescing to form the superior trunk, which can then be visualized distal to the convergence of the C5 and C6 nerve roots but proximal to the take-off of the suprascapular nerve [1].

**Figure 1:**
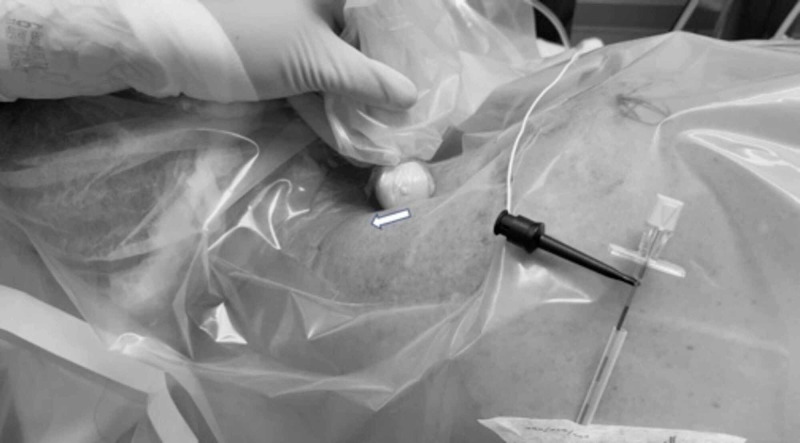
Cephalad rotation of the lateral edge of the ultrasound probe while maintaining the sonographic view of the upper trunk.

We use either of the scanning technique as described above. We recommend scanning the area of interest, caudad to cephalad, few times, to confirm the coalesce of the C5-C6 nerve root. Once the upper trunk and the surrounding structures are identified, the lateral edge of the linear probe is rotated in anticlockwise direction to steer the needle entry site away from the shoulder (Figure 1).

**Figure 2:**
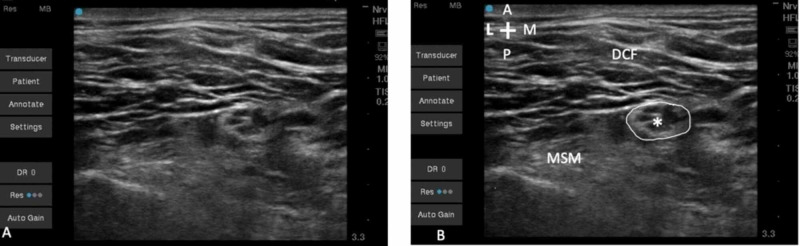
(A) Unlabeled view of the ultrasound view of the upper trunk with surrounding anatomy. (B) The C5 and C6 roots coalesced into the upper trunk (*), which has a well-defined hyperechoic boundary (circled). DCF, deep cervical fascia; MSM, middle scalene muscle

The needle is advanced in-plane to the ultrasound beam in a lateral-to-medial direction through the middle scalene muscle (Figure 2). At this location, the dorsal scapular nerve and or long thoracic nerve has most likely already exited off the C5 nerve root, but a combination of ultrasonographic visualization and neurostimulation with an initial current of 2.0 mA, pulse width of 100 ms, and a frequency of 2 Hz may be used to eliminate this possibility [4].

**Figure 3:**
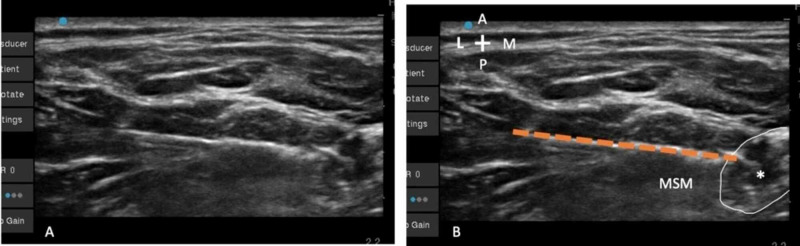
(A) Unlabeled view of the Tuohy needle approaching the upper trunk. (B) The needle (interrupted orange line) is seen approaching the upper trunk (*) in-plane under the deep cervical fascia through the MSM with its tip is adjacent to the lateral aspect of the upper trunk. MSM, middle scalene muscle

Once the needle tip is in proximity to the upper trunk, a contraction of the biceps is sought (Figure 3). We prefer that the needle traverse through the middle scalene muscle as it provides more stability to the catheter, and any retrograde spread of local anesthetic along the catheter is likely to spill into the middle scalene muscle rather than the subcutaneous fascial plane continuous with the deep cervical fascia containing the phrenic nerve. While there is controversy regarding which twitch elicited by a nerve stimulator (biceps or deltoid) is superior [6-8], in our experience, pursuing the twitch of biceps results in almost a complete block of the brachial plexus barring the lower trunk.

**Figure 4:**
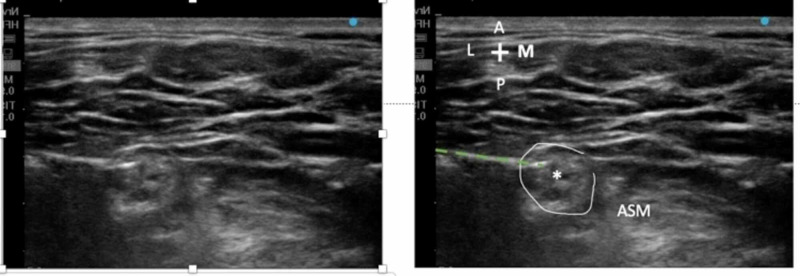
(A) Unlabeled view of the perineural catheter and upper trunk. (B) The perineural catheter (interrupted green line) is seen inside the upper trunk (*), again seen surrounded by a well-defined fascia (circled). ASM, anterior scalene muscle

A stimulating catheter is then advanced such that the biceps twitch is preserved throughout (Figure 4). We almost always find that the needle at this juncture is in the middle of the upper trunk, most likely in between its anterior and posterior divisions, making this technique extremely reproducible [3]. The presence of rich connective tissue in the upper trunk, which was demonstrated by Aguirre et al. in histological preparations, can be seen clearly by ultrasound [2]. We do not recommend overshooting the target, as advancing the needle medially past the upper trunk will increase the chances of catheter displacement. The catheter is threaded at least 5 cm within the neural tissue. The catheter placement is confirmed by conducting the Raj test followed by a bolus of 10 mL of 0.2% ropivacaine and an infusion at 5 mL/hour and a PCRA (patient-controlled regional anesthesia) bolus of 5 mL every 1 hour.

Ultrasound-guided upper trunk block is considered a safer but equianalgesic alternative to interscalene block [4]. Cros Campoy et al. injected 5 mL radiolabeled dye between the anterior and posterior divisions of the upper trunk of two fresh cadavers and found that it stained not only the entire upper trunk along with the suprascapular nerve but also the C5 and C6 roots [3]. The middle trunk was also found to be partially stained, with no evidence of dye staining the lower trunk, anterior aspect of the anterior scalene muscle, or the phrenic nerve [3]. A brachial plexus sonoanatomy report determined that the upper, middle, and lower nerve trunks stay together in the interscalene region, and the spread of the neural elements at the two levels recorded were no different from one another, implying that a nerve block can be adequately performed at a range of levels within the interscalene groove and not just the classical position at the level of the C6 vertebra [9].

Microanatomical studies have revealed that the relative and absolute amount of the brachial plexus nonneural tissue increases proximal to distal, even though the amount of neural tissue remains about the same throughout the brachial plexus [10]. This makes the upper trunk an ideal location to place a nerve block catheter since the non-neural tissue allows for easy passage of the nerve block catheter. Moreover, a recent study comparing interscalene and upper trunk single-injection block found that hemidiaphragmatic paresis was observed in 97.5% of the interscalene block group versus 76.3% of the superior trunk block group; however, complete paresis was observed in 72.5% versus 5.3% of the patients, respectively [4].

## Conclusions

Many patients who receive an interscalene catheter can suffer from incomplete or complete blockade of the phrenic nerve despite a successful block. A single injection of the upper trunk block seems promising in preventing complete paralysis of the hemidiaphragm. Our technique illustrates that a perineural catheter can be placed safely within the upper trunk, thus extending the analgesia. We noticed no perioperative complications, and hope that the present case series will lead to further studies investigating the utility of the upper trunk perineural catheters and not just a single injection alone.
